# Native Hyperaccumulator Plants with Differential Phytoremediation Potential in an Artisanal Gold Mine of the Ecuadorian Amazon

**DOI:** 10.3390/plants11091186

**Published:** 2022-04-28

**Authors:** Irene Chamba-Eras, Daniel M. Griffith, Carolina Kalinhoff, Jorge Ramírez, Manuel Jesús Gázquez

**Affiliations:** 1 Departamento de Química, Universidad Técnica Particular de Loja, San Cayetano Alto s/n, Loja 1101608, Ecuador; jyramirez@utpl.edu.ec; 2 Departamento de Ciencias Biológicas y Agropecuarias, Universidad Técnica Particular de Loja, San Cayetano Alto s/n, Loja 1101608, Ecuador; dgriffith@utpl.edu.ec (D.M.G.); cgkalinhoff@utpl.edu.ec (C.K.); 3 Departamento de Física Aplicada, Escuela Superior de Ingeniería, Universidad de Cádiz, Campus de Puerto Real avenida, República Saharahui s/n, 11510 Puerto Real, Spain; manueljesus.gazquez@uca.es

**Keywords:** phytoremediation, heavy metals, *Erato polymnioides*, *Miconia* sp., bioaccumulation and translocation factors

## Abstract

In tropical forests of southern Ecuador, artisanal gold mining releases heavy metals that become xenobiotic with indefinite circulation and eventual bioaccumulation. Restoration and rehabilitation of degraded mining sites represent a major ecological, technological and economic issue. In this study, we estimate the capacity of two native woody plants to accumulate cadmium (Cd), lead (Pb), zinc (Zn) and mercury (Hg), with the goal of developing effective strategies for phytoremediation of mining sites. Individuals of *Erato polymnioides* and *Miconia* sp., as well as their rhizospheric soils, were sampled from a natural zone (NZ) of montane cloud forest, used as a control, and a polluted zone (PZ) subjected to active gold mining. Concentrations of the four heavy metals were analyzed using atomic absorption spectrophotometry. Cd, Zn and Hg concentrations were higher in soils of PZ than NZ. Bioaccumulation (BCF) and translocation factors (TF) showed that *Miconia* sp. has potential for Cd and Zn phytostabilization, *E. polymnioides* has potential for Cd and Zn phytoextraction, and both species have potential for Hg phytoextraction. Despite the low productivity of these species, their adaptability to the edaphoclimatic conditions of the region and the possibility of using amendments to increase their biomass could compensate for the effectiveness of these species in reclaiming soils contaminated by mining.

## 1. Introduction

Given their capacity to bioaccumulate in the food chain, heavy metals are of great concern due to their long-term effects on human health, especially in developing countries [1,2]. Mining and metal milling operations are recognized as among the principal sources of heavy metal contamination of soil, water and air [3,4]. Artisanal mining, which refers to informal mining activities carried out with low technology and minimal machinery, is particularly destructive because it is so widespread. Occurring in approximately 80 countries, mainly in the Global South, artisanal mining is practiced by an estimated 100 million people and supplies roughly 20% of the world’s minerals and metals [5,6]. Increasing public awareness of the impacts of artisanal mining on ecosystems and human health has stimulated interest in innovative technologies to remediate the contaminated wastelands that result from this form of mining.

Artisanal mining is widespread in South America, occurring primarily in Bolivia, Venezuela, Colombia, Brazil, Peru and Ecuador. In the Andean region, it is often carried out in previously undisturbed ecosystems such as paramos, cloud forests and rain forests, where it represents a threat to the flora, fauna and local communities living downstream [7]. The most common technique used for small-scale gold mining (SSGM) is amalgamation, which has dramatically increased the release of mercury into the environment over the last fifty years [8]. As a result, mercury has become a major pollutant of surface and groundwater in many localities [9].

In the southern Ecuadorian Amazon, heavy metals like Pb and Zn that are naturally present in polysulfides and metamorphic rocks are often released into the environment during both SSGM and large-scale multi-metallic exploitation [10]. As a co-product of Zn extractive metallurgy, Cd has become another major contaminant. The magnitude and complexity of the pollution caused by these xenobiotic elements are due to their indefinite circulation and eventual bioaccumulation [11].

The severe impacts that heavy metals render on human health and the environment underscore the need for methods and tools to collect and remove these contaminants from polluted soils. Physical methods for soil remediation include soil substitution, thermal desorption, membrane filtration and ion exchange, while chemical methods include chemical precipitation, chemical leaching, chemical fixation and immobilization [12]. However, both physical and chemical methods are generally costly, cause irreversible changes in the soil, and result in secondary contamination [13,14]. In contrast, biological methods for soil remediation, known collectively as bioremediation, generally consist of cost-effective and environmentally sustainable processes that detoxify heavy metals in contaminated soils and water bodies [15,16] through the use of bacteria, fungi, plants or a combination of these organisms [12].

Phytoremediation is a type of bioremediation that utilizes plants to reduce the toxic effects of heavy metals in the environment [17]. As an emerging alternative technology to conventional remediation approaches, phytoremediation offers the advantage of being economically and ecologically sustainable [18,19,20]. Among the phytoremediation technologies applicable to soils contaminated with heavy metals, two of the most commonly used are phytoextraction and phytostabilization [21]. In phytoextraction, rapidly growing plants that tolerate high concentrations of metals in their aerial tissues are used. In phytostabilization, plants that possess a strong ability to reduce metal mobility in the rhizosphere or roots are used [22,23].

Phytoextraction is considered a permanent solution for the removal of heavy metals (assuming it includes the final disposal of aerial biomass), unlike phytostabilization, which retains metals underground [21]. High aboveground biomass production, high tolerance, and the ability to extract, transfer and accumulate metals are crucial for the success of phytoextraction. In this regard, accumulator plants with high biomass production, known as hyperaccumulator plants, are the most suitable for phytoremediation. Hyperaccumulator plants can exceed 100 or times more the normal concentrations of accumulated metals or metalloids in their aboveground biomass without showing signs of phytotoxicity, although in some cases they can have low productivity [24]. For this reason, it is generally accepted that species used in phytoremediation with high biomass production capacity can compensate for their relatively low metal accumulation capacity [25]. Phytoextraction potential can be estimated by calculation of the bioconcentration factor (also known as the bioaccumulation factor or biological absorption coefficient) and translocation factor. The bioconcentration factor (BCF) is defined as the ratio of the total concentration of an element in harvested plant tissue to its concentration in the soil where the plant was growing, and the translocation factor (TF) is defined as the ratio of the total concentration of an element in the aerial parts of the plant to its concentration in the roots [22].

In Ecuador, the exploitation of minerals through both large-scale and artisanal mining has increased substantially in recent decades, with serious repercussions for the environment and human health. Conventional remediation techniques have been shown to be effective, but their high construction and operation costs make these methods untenable in regions with limited technological and financial resources. Therefore, it is necessary to develop new, cost-efficient alternatives to clean contaminated mining sites and improve the health of the affected population and environment. Research has shown that certain plants that grow naturally in mine tailings can be used for in situ soil decontamination [26,27,28]. However, there are few studies that have evaluated the phytoremediation potential of native species that grow spontaneously in mining areas of Ecuador. The main objective of this study was to evaluate the potential for heavy metal accumulation of two plant species that grow abundantly around the gold mines of the Chinapintza Mining District in southern Ecuador: *Erato polymnioides*, which was determined to be a hyperaccumulator for mercury (Hg) in this zone [6], and *Miconia* sp., which grows spontaneously in areas heavily disturbed by mining. Specifically, we: (1) determined the concentration of heavy metals in vegetative organs and rhizospheric soils; (2) calculated the bioaccumulation and translocation factors in order to propose phytoremediation strategies for the region; and (3) estimated the productivity of each species.

## 2. Results

### 2.1. Metal Concentration in Soils

Mean (± SD) concentrations of the four heavy metals in soil are shown in Table 1. The relative error, which represents the standard deviation relative to the mean [29], is related to the accumulation capacity of these heavy metals for the plants analyzed.

As expected for mining areas, metal concentrations in soils were highly heterogeneous, especially in the polluted zone (PZ), where the relative errors of the means ranged from 12–60%, reaching a maximum for Hg in the rhizospheric soil of *Miconia* sp. Heterogeneity was lower in the natural zone (NZ), where relative errors of the means ranged primarily between 20–40%, with the exception of Cd and Zn for *E. polymnioides*.

Plants of *Miconia* sp. were exposed to mean soil concentrations of Cd, Zn and Hg that were 2.7 to 4.6 times higher in PZ than those in NZ. Soils around *E. polymnioides* exhibited concentrations of the same elements that were 3.5 to 12.3 times higher in PZ relative to NZ. The exception in both cases was Pb, which varied little between zones. Soils in NZ exhibited lower concentrations of Cd, Pb and Hg for *E. polymnioides* than *Miconia* sp., while Zn concentration for *E. polymnioides* was double that observed for *Miconia* sp. The rhizospheric soils of *E. polymnioides* in PZ showed substantially higher concentrations of all metals than those of *Miconia* sp. except Pb.

### 2.2. Metal Concentration in Plants

**Figure 1:**
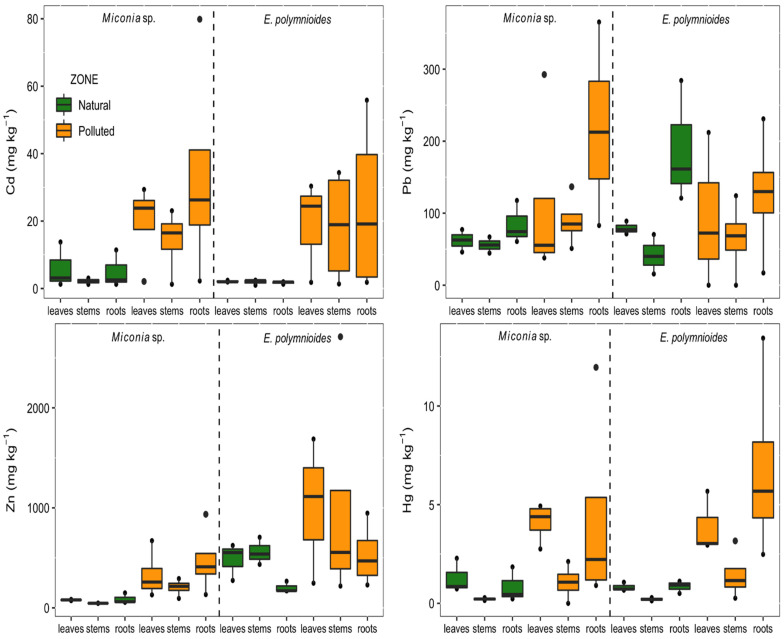
Concentration of heavy metals (mg kg^−1^) in leaves, stems and roots of *Miconia* sp. and *E. polymnioides* in a natural zone and polluted zone of southern Ecuador.

In *Miconia* sp. plants, all four metal concentrations were significantly higher in PZ than NZ, but none were significantly different between leaves, stems and roots (Figure 1; Supplementary Table S1). In *E. polymnioides* plants, the concentration of Cd and Hg was significantly higher in PZ than NZ. Hg was also significantly higher in leaves and roots than stems of this species, while Zn was significantly higher in leaves and stems than roots. Like *Miconia* sp., no differences in Cd were observed between plant parts in *E. polymnioides*, but the maximum concentrations reached in PZ were 55 and 40 mg kg^−1^ in roots and leaves, respectively. Hg reached a maximum value of 13 mg kg^−1^ in *E. polymnioides* roots, while Zn reached nearly 1000 mg kg^−1^ in this species’ leaves in PZ.

The total weight and biomass distribution among plant parts of the two species be-tween the zones are shown in Table 2. For *Miconia* sp., total weight was 11 and 2 g in NZ and PZ, respectively. For *E. polymnioides*, total weight was 52 and 18 g in NZ and PZ, respectively. Biomass distribution between leaves, stems and roots of *Miconia* sp. ranged from 39–41, 30–45, and 10–30% in NZ, respectively. However, these percentages were different in PZ, where the biomass found in leaves constituted 60% of the total mass of the plant, whereas that of stems and roots dropped to 20–25% and 14–19%, respectively.

In *E. polymnioides*, the relative contribution of each part was similar between the zones. The contribution of roots was between 10–30% in PZ and 5–10% in NZ. Stems showed values of 75–80% in NZ and 40–80% in PZ, while leaves represented between 15–35% in both zones.

### 2.3. Bioaccumulation and Translocation Factors

For both species, the bioaccumulation factor (BCF) of Cd, Zn and Hg were greater than 1, with the exception of the BCF for stems (Table 3). In the case of Pb, the only BCF close to one (0.80) was that of *E. polymnioides* roots in PZ. The translocation factor (TF) of leaves/roots were greater than or very close to 1 for Cd, Zn and Hg in both species.

For *Miconia* sp., BCF of Cd, Pb and Zn in PZ was higher in roots than stems and leaves, while BCF of Hg was higher in leaves (Table 3). The highest BCF value for this species was observed for Cd in roots in PZ (6.50). When comparing BCF between NZ and PZ, we observed that BCF of Cd, Pb, Zn and Hg in *Miconia* sp. leaves and stems changed very little, while that of roots doubled for all metals except Hg. TF of *Miconia* sp. leaves/roots of was higher than that of stems/roots, and a decrease in PZ relative to NZ was observed for the four metals evaluated. The highest TF value observed for this species was for Hg in leaves/roots (2.53). For Cd, Pb, and Zn, the highest TFs were 1.14, 0.75, and 1.00, respectively (Table 3).

For *E. polymnioides*, higher values of BCF were also observed for Cd in PZ, with BCF in leaves (6.19) being very close to the BCF in roots (5.64). Only for Cd and Pb was an increase in BCF observed in PZ compared to NZ (Table 3). The values of BCF for Pb and Zn in *E. polymnioides* were generally higher than in *Miconia* sp. (Table 3). As in *Miconia* sp., TF leaves/roots were higher than TF stems/roots in *E. polymnioides* and, in general, TF values in NZ with respect to PZ increased or remained the same. The highest TF values observed for *E. polymnioides* were stems/roots (2.79) and leaves/roots (2.40) for Zn. For the metals Cd, Pb, and Hg, the highest TFs were 1.55, 0.53, and 1.27 leaves/roots TF, respectively (Table 3).

### 2.4. Tolerance Index (TI) of Plant Yield

The tolerance index was evaluated based on the biomass yield of each species, projecting a planting of 10,000 plants per hectare. For *Miconia* sp. we estimated a total of 108,800 g plants ha^−1^ in NZ, and 19,600 g plants ha^−1^ in PZ. For *E. polymnioides*, we estimated 523,200 g plants in NZ and 186,300 g plants in PZ. The analysis yielded a TI of 0.18 for *Miconia* sp. and 0.36 for *E. polymnioides*.

## 3. Discussion

Soils of both the natural and polluted zones contained high concentrations of the four metals evaluated according to the Ecuadorian Environmental Technical Standard for soils. According to this standard, allowable limits for Cd, Pb, Zn and Hg are 2, 100, 200 and 0.8 mg kg^−1^, respectively [30]. These limits were exceeded by all four metals in soils of the polluted zone and by Pb and Hg in soils of the natural zone. High concentrations in the substrates that support the native forest of the area could be a natural occurrence, given the existence of numerous natural polymetallic deposits throughout the region [31]. On the other hand, mining exploitation has occurred since pre-colonial times [32], so it is plausible that our reference ecosystem corresponds to a secondary forest established in a formerly mined area. Regardless of the reasons for the high Pb and Hg levels, the concentrations of Cd, Zn and Hg were substantially lower in soils of the natural area compared to the currently mined area.

Soil concentrations of Cd and Zn detected in the PZ of our study were similar to those reported in soils contaminated by mining in the same area with values of 5 and 650 mg kg^−1^, respectively [33]. In contrast, Pb concentrations were roughly half (430–523 mg kg^−1^ in our study vs. 1000 mg kg^−1^ in [33]) and Hg concentrations were twice those reported previously from the area (6.1–10.2 mg kg^−1^ in our study vs. 4.8 mg kg^−1^ in [6]). It should be emphasized that the amalgamation process is used extensively to mine gold in the region (i.e., Zamora-Chinchipe), which generates an Au-Hg alloy that is then heated in open vessels to separate these metals, yielding volatilized Hg. Given that the region experiences high rainfall throughout much of the year, Hg eventually accumulates in the soil [8] and can move into river systems [34]. The variability found in Hg and the other metals was likely a result of the haphazard nature of mining operations in the polluted zone, as there is currently no specific waste collection or treatment plan for this area.

Cd levels detected in *Miconia* sp. and *E. polymnioides* plant tissues were high in comparison with other studies of heavy metal contamination in plants growing in mining areas. For example, average Cd concentration in plants growing in the polluted zone varied between 20 and 40 mg kg^−1^, which is more than a magnitude higher than that found in *Artemisia vulgaris* (0.62 mg kg^−1^) from mining areas in Vietnam [35] and *Datura inoxia* (0.44 mg kg^−1^) from artisanal mines in Nigeria [36]. Regarding Hg, the mean concentrations detected in both species (2.0 and 7.0 mg kg^−1^) were similar to those observed for most dicots growing on acid mine tailings containing heavy metals in Niger [37]. They were also similar to values reported in forage plants from artisanal mining areas in Indonesia (9.90 mg kg^−1^) [38].

Contrary to Cd and Hg, Pb and Zn concentrations in the two study species were lower than those reported for plants from other mining areas. *Stipa capensis* growing on abandoned mine sites in Morocco accumulated 282.3 mg kg^−1^ Pb in its roots [38], while *Miconia* sp. and *E. polymnioides* from the same area of our study showed lower Pb values. In *Miconia* sp., Pb concentrations of 400, 100 and 150 mg kg^−1^ were observed in roots, stems and leaves, respectively, while in *E. polymnioides*, Pb was 200 mg kg^−1^ in leaves [33]. Similarly, we found Zn levels for *Miconia* sp. and *E. polymnioides* to vary between 50 to 1500 mg kg^−1^. These values are low compared to species growing spontaneously on extremely contaminated acid mine tailings in Spain, where Zn levels in stems varied from 94 mg kg^−1^ in *Erica arborea* to 3391 mg kg^−1^ in *Coincya monensis* [38]. A high Zn concentration of 2938 mg kg^−1^ was also reported in *Reseda lutea* plants growing near a Pb-Zn mine in Iran [35]. Thus, while Cd was high in the tissues of our study species, Hg was similar, and Pb and Zn were lower compared to other plants growing on mine sites.

Bioaccumulation (BCF) and translocation factors (TF) showed that *Miconia* sp. possessed a high capacity for Cd and Zn phytostabilization, whereas *E. polymnioides* had high capacity for Cd and Zn phytoextraction. In *Miconia* sp., BCF indicated a high tolerance for Cd in the polluted zone with the potential to accumulate 6.5 times more of this metal in its roots than the immediate soil. Similarly, *Miconia* sp. accumulated 2.3 times more Zn in its roots than the soil in the polluted zone. The fact that TF was less than one in this zone indicating metal accumulation in the roots, in addition to the large increase in BCF from the natural to polluted zone in the roots relative to leaves and stems, supported classification of *Miconia* sp. as a potential phytostabilizer of Cd and Zn [33]. In contrast, *E. polymnioides* accumulated Cd to the same degree in roots and leaves in the polluted zone. This result, along with TF > 1 in both zones indicating metal accumulation in the aerial parts of the plant, supported classification of this species as a potential Cd phytoextractor. BCFs of Cd in *E. polymnioides* reported in previous studies from the same area were also greater than 1, with values of 1.10 and 1.68 for roots and leaves, respectively [28]. Notably, BCFs of Cd found in our study were high compared to those reported for other species such as *Stipa tenacissima* growing in Pb-Zn mining areas in Morocco (2.72) [35]. *E. polymnioides* also exhibited bioaccumulation of Zn in sufficient proportions and TF > 1 in both zones to be considered a phytoextractor of this metal. BCFs that we found for Zn were high relative to those for *Artemisia herba alba* in Morocco, which had a maximum of 1.69 [35], as well as *E. polymnioides* roots (1.64) and leaves (1.40) from the same polluted zone [34]. Evidence thus suggests a high potential of *Miconia* sp. for Cd and Zn phytostabilization and a high potential of *E. polymnioides* for Cd and Zn phytoextraction.

Hg accumulation in plant roots is reported to be a defense mechanism that hinders phytoextraction of this highly toxic metal [39]. Low amounts of Hg in roots relative the surrounding soil [39] suggest a reticence on the part of some plants to absorb this metal. In this context, the search for native plants with the ability to bioaccumulate and transfer mercury to the stem has become a priority in phytoremediation efforts, especially in economically emerging countries suffering from Hg contamination. Our results confirm the ability of *E. polymnioides* to accumulate mercury in its roots as reported by [33]. Unlike that study, however, we also found BCF > 1 for leaves in the polluted zone, which, together with evidence for Hg accumulation in the leaves relative to the roots (TF > 1), suggests that *E. polymnioides* has potential for phytoextraction of this metal. The evidence for *Miconia* sp. as an effective candidate for Hg phytoextraction was even stronger, given a higher TF in leaves and thus greater efficiency in translocating Hg to its aerial organs than *E. polymnioides*. Further research conducted under controlled conditions (e.g., greenhouse) is necessary to assess how these attributes change between varying levels of contamination and at different growth stages of each species.

Neither study species showed a capacity for phytostabilization or phytoextraction of lead. All BCFs of Pb were less than one and lower than those reported by previous studies, for example, BCF was 2.67 in *A. herba alba* [35] and 1.20 in *Zea mays* [40]. Moreover, the TF of both species was less than one in the natural and contaminated zones. According to the Ecuadorian Environmental Technical Standard for soils, Pb concentration in the study area was 3 to 5 times higher than the recommended limit. However, we only measured total concentrations and not bioavailable fractions. It is possible that much of the lead present in the soil cannot be taken up by plants. Lead’s low bioavailability limits its uptake from the soil, reducing the effectiveness of phytoextraction [21]. On the other hand, the higher Pb concentration in the roots of both species suggests the possibility of phytostabilization of the bioavailable fraction of the metal, especially in *E. polymnioides*, whose BCF of Pb in roots was 0.8. Studies are needed to determine how limited bioavailability affects the capacity of these species to phytostabilize and phytoextract Pb and other heavy metals.

Accumulators are plant species that tolerate heavy metal concentrations above the limits established by national and international regulations and meet the criteria of both BCF and TF > 1. According to [41], the metal hyperaccumulation limits in plant shoots for Zn, Pb, Cd and Hg are 10,000, 1000, 100 ppm and 10 ppm, respectively. According to these criteria, *E. polymnioides* can provisionally be considered a hyperaccumulator of Hg, although thresholds for Hg need to be more clearly established. Currently, plants that concentrate more Hg in their stems have been defined as potential accumulators, but in most, BCF < 1 [42]. On the other hand, unlike hyperaccumulator plants, which concentrate large amounts of metals in their biomass, accumulator plants concentrate less but compensate with higher biomass production [42]. Thus, in addition to tolerating heavy metals, one of the most important conditions for phytoextraction is that the accumulator plant produces enough biomass to accumulate metals to the same degree as that of a hyperaccumulator plant [43]. To fully assess the phytoremediation capacity of a potential accumulator species, it is necessary to estimate its productivity through controlled experiments and field data to verify the overall heavy metal uptake.

Despite the limitations of field sampling, this study contributes evidence that *Miconia* sp. and *E. polymnioides* have high potential for phytoremediation and could be incorporated into regional strategies for bioremediation of mining liabilities in Amazonian forests. In mining areas throughout the world, high values of Cd, Zn and Hg translocation have been reported for various species, indicating their potential use as phytoextractors. For example, TF for Cd of 1.69 and TF for Zn of 1.9 were observed in *Euphorbia hyssopifolia* and *Pueraria montana* in gold mining areas in Ghana, respectively [22,44]. In an artisanal mining area in Indonesia, TF for Hg of 0.84 was observed for a native *Guava* sp. [45], and in a mining area of southeast China, TF for Hg of 2.62 was observed for the native species *Cyrtomium macrophyllum* [46].

Although total plant weight and the relative weights of plant organs are generally not used as criteria to evaluate species’ capacity for phytoremediation, we consider these parameters important because they provide a clearer picture of how contaminants are distributed within the plant. The percent weight of leaves in *Miconia* sp. and of leaves and roots in *E. polymnioides* increased from NZ to PZ. This could be important to help determine the best phytoremediation strategies for metals with BCF > 1 in these organs, as was the case for Cd, Zn and Hg, because large proportional biomass coupled with large BCF of the organ implies greater accumulation of the metal in question and thus, in effect, more efficacious phytostabilization or phytoextraction.

Total plant weight also allowed us to calculate the tolerance index (TI), which provided a rough indicator of the level of stress experienced by plants growing in the polluted zone relative to the natural zone. TI was substantially less than one for both study species, which suggests high levels of stress in the mining area. By comparison, TI values reported for grasses and legumes that became highly resistant to ash from bituminous combustion were higher than those in our study [47], which suggests that resistance to contaminants could develop over time. The higher TI of *E. polymnioides* implies that this member of the Asteraceae family was more productive than *Miconia* sp. in the polluted zone, which may be due to higher tolerance of heavy metals and/or resistance to the continual disturbance caused by mining activities. Several Asteraceae species have been recommended for phytoremediation of pollutants, such as Fe, Cd, Hg, Cr, As, Ni, Cu, Cd, Co, Mn, Pb, Cr, Zn, polycyclic aromatic hydrocarbons, and radionuclides [48]. The higher TI of *E. polymnioides* implies a more effective capacity than *Miconia* sp. for phytoremediation in the contaminated zone, although strategies combining the two species should not be ruled out.

## 4. Materials and Methods

### 4.1. Study Area

**Figure 2:**
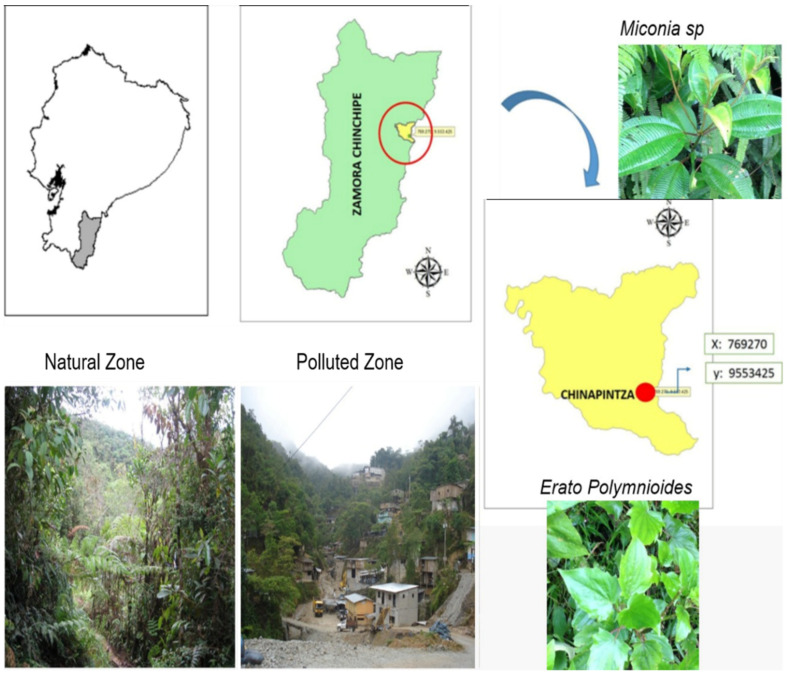
Location of the study area and pictures of the two studied zones and two native species selected for study.

This study was carried out in Zamora-Chinchipe Province in southern Ecuador, where approximately 23% (282,998 ha) of the total area is dedicated to artisanal mining [49]. The study site was located near the settlement of Chinapintza, which is adjacent to the Peruvian border in the Condor Mountain range (402016S, 7834014W; Figure 2). Characterized by rugged terrain and a high incidence of geological faults, the study site was centered on an active mining settlement located at an elevation of 1854 m.a.s.l. and sur-rounded by tropical montane cloud forest. We identified two zones at the site: (1) a natural zone (NZ) consisting of secondary forest located upslope from the mining area and free of the influence of mining activity; and (2) a highly degraded polluted zone (PZ) subjected to continuous artisanal mining of gold and other precious metals from stream sediments and banks. This latter zone was under constant change from mining activities such as soil excavation, stream channel modification, and construction of paths and infrastructure, which caused persistent disturbance and severely limited regeneration of the native vegetation.

### 4.2. Plant and Soil Sampling

We selected two native plant species that were relatively abundant in both NZ and PZ for assessment as potential heavy metal hyperaccumulators: *Erato polymnioides* DC. (Asteraceae) and *Miconia* sp. (Melastomataceae). *Miconia* sp. represents a single morphospecies that did not exhibit fertile characters at the time of sampling, so we were unable to identify it to species level. Despite this limitation, we selected it for study due to the high number of juvenile individuals found in both zones relative to other species.

Seven individuals of *E*. *polymnioides* and *Miconia* sp. were collected in each zone. In addition, we collected the soil around the base of each individual, which included the roots and rhizosphere. Sample sizes were limited due to the low number of individuals of both species in PZ, presumably due to continued disturbance of the area from mining activities. According to the recommendations of [50], sampling took place far from active roads and the surface of fresh plant material was checked to be free of dust.

### 4.3. Plant and Soil Analysis

In the laboratory, plant samples were washed with ultra-pure water (Merck Millipore Milli-Q, Darmstadt, Germany), placed in paper bags, and dried in an oven at 50 °C for one week. Soil samples (0.5–1.0 kg each) were dried at 50 °C until the weight was constant. Dried samples of both plant tissues and soil were weighed and mechanically grounded using a stainless steel grinder (particle diameter 100 µm) for digestion. Individual plants were divided into leaves, stems and roots. Therefore, there were 7 replicates of 2 species from 2 zones divided into 4 parts (3 plant organs + 1 soil sample). In total, 112 samples were processed to determine heavy metal concentrations.

Subsamples (~0.2 g for plants and ~1 g for soil) were weighed for digestion and subsequently added to a mixture of HCl and HNO_3_ in a 3:1 ratio (*v/v*) (aqua regia). Considered effective for measuring the “total” amount of trace elements in soils, the aqua regia digestion method (USEPA 3050 or ISO standard 11466) provides an estimate of the maximum availability of elements to plants [51]. Samples were left for 1 week to soak in the acid, after which they were digested in an open heat block (Environmental Express 54 HotBlock SC154) for 2 h. After cooling, samples were diluted to 100 mL with 0.1 M HCl and stored until the metal concentration analyses.

Upon filtering the sample solutions through filter paper, concentrations of heavy metals in the digested solutions were analyzed immediately using an atomic absorption spectrophotometer (Perkin-Elmer, AANALYST 400, Akron, OH, USA). The respective wavelength (nm), precision measured as relative standard deviation (%), and detection limit (mg kg^−1^) of the elements studied were as follows: 283.31, 1.0 and 0.05 for Pb; 213.86, 2.31 and 0.005 for Zn; and 228.80, 1.7 and 0.002 for Cd. Reproducibility of the method used for digesting the leaf samples was verified using triplicate analyses [52].

We applied the hydride generation technique coupled to an AA using an electrodeless discharge lamp to determine total Hg concentration in the samples [53]. A Hg standard calibration curve (100, 200, and 300 µg L^−1^) was prepared in 10 mL of acid mixture containing 1.5% HNO_3_ by triplicates. We also ran two blank samples simultaneously to estimate the background metal contamination from the digestion procedure. For each sample, 10 mL of an acid mixture containing 1.5% HNO_3_ were added to 5 mL of the digestion mixture (prepared by triplicates). Hg was determined using an aqueous solution of 3% (*w/v*) NaBH4 in a 1% (*w/v*) NaOH solution that was freshly prepared and filtered as a reducing agent. Analytical grade chemical reagents and highly purified deionized water were used throughout the process.

Accuracy of the analytical methods was verified based on certified reference materials and standard solutions: CRM029-50G Trace Metals—Sewage Sludge 2 (RT Corporation, 2931 Soldier Springs Rd-USA, Tokyo Japan) and CRM027-50G Trace Metals—Sandy Loam 10 (RT Corporation-2931 Soldier Springs Rd-USA, Tokyo Japan).

### 4.4. Calculation of Bioaccumulation and Translocation Factors

To evaluate the potential accumulation of heavy metals in the study plants [54], the bioaccumulation factor (BCF) was calculated as the ratio of the trace metal concentration in plant tissue to that in soil (BCF = C_plant_/C_soil_), where C_plant_ and C_soil_ are the concentrations given in units of mg kg^−1^ of dry weight.

To evaluate the capacity of the plants to transfer heavy metals from the soil to aerial parts of the plants, the translocation factor (TF) was calculated as the ratio of metal concentration in the aerial parts to that in the roots: TF = C_aerial_/C_roots_, where C_aerial_ and C_roots_ are the concentrations given as mg kg^−1^ of dry weight in aerial parts of the plant and root, respectively [55]. TF values < 1 indicate metal accumulation in the roots while values > 1 indicate accumulation in the aerial parts of a plant.

Thus, concerning the aforementioned, the following criteria apply: if TF and BCF > 1, phytoextraction occurs; if TF < 1 and BCF > 1, phytostabilization is attained [35].

### 4.5. Calculation of the Tolerance index (TI)

Finally, we calculated the tolerance index, which is commonly employed to evaluate the effect of various types of stress on plant growth and yield, as TI = Y_Tr_/Y_Ct_ [56]. *Y_Tr_* and *Y_Ct_*, where _Tr_ stands for treatment and _Ct_ for control, are measured in units of g ha^−1^ DM and represent the total yield of plants growing in PZ and NZ, respectively. It was estimated that 10,000 plants could be planted per hectare as a phytoremediation strategy.

### 4.6. Statistical Analysis

The effect of zone (natural vs. polluted) and plant part (leaves, stems, roots) on the concentration of the four metals was analyzed separately for *Miconia* sp. and *E. polymnioides* with linear models. Given that the interaction between zone and plant part was not significant for any of the metals in either species, we analyzed only the main effects of these factors. Structures allowing for different variances by zone or the interaction between zone and plant part were included in the models to account for within-group heteroscedasticity [57]. Residuals were examined graphically in the final models to ensure that assumptions of normality and homogeneity of variance were met. Tukey’s HSD tests were used to make multiple comparisons between zones or plant parts when these variables were significant in the final model (*p* < 0.05). Linear models and Tukey tests were implemented using the *nlme* [58] and *emmeans* [59] in R version 4.1.3, respectively [60].

## 5. Conclusions

As in areas throughout southern Ecuador, mining activities in Chinapintza have produced massive deposits of inadequately managed hazardous waste. This study found that the concentration of Cd, Pb, Zn and Hg in soils of the area exceeded the regulatory thresholds of Ecuador, thereby constituting a serious threat to human health and the environment. Our findings provide new insights into the tolerance of two woody plants native to tropical montane cloud forest, *Erato polymnioides* and *Miconia* sp., to xenobiotic heavy metals. High bioconcentrations of Cd, Zn and Hg were reported in situ, mainly in roots and leaves, and two of the phytoextraction criteria (BCF and TF > 1) were met for Cd, Zn and Hg. Specifically, evidence showed that *Miconia* sp. has potential for Cd and Zn phytostabilization, *E. polymnioides* has potential for Cd and Zn phytoextraction, and both species have potential for Hg phytoextraction. Neither species met the standards to be considered hyperaccumulators of Cd, Pb or Zn; however, some individuals of *E. polymnioides* exhibited Hg concentrations > 10 ppm in their roots, which suggests that this species could be a hyperaccumulator of Hg. Although the projected productivity of these species was not high, their adaptation to the edaphoclimatic conditions of the region and the possibility of using amendments to increase their biomass, could compensate for the effectiveness of these species for the reclamation of soils contaminated by mining.

## Figures and Tables

**Table 1:** Mean concentration of cadmium (Cd), lead (Pb), zinc (Zn) and mercury (Hg) (mg kg^−1^) in soils of the two study species in a natural zone (NZ) and polluted zone (PZ) of an active mining area in southern Ecuador. Standard deviations and relative errors (%) are shown. NZ was a tropical montane forest located upslope from the mining area and free from contamination, whereas PZ was subjected to continuous artisanal mining of gold and other precious metals from stream sediments and the adjacent soils.

		Cd	Pb	Zn	Hg
*Miconia* sp.	NZ	1.62 ± 0.59 37%	510 ± 150 29%	85 ± 16 19%	1.62 ± 0.54 33%
	PZ	4.44 ± 1.68 38%	523 ± 63 12%	390 ± 140 36%	6.1 ± 3.6 60%
*E. polymnioides*	NZ	0.42 ± 0.24 57%	326 ± 75 23%	169 ± 93 55%	1.20 ± 0.22 18%
	PZ	5.15 ± 2.23 43%	430 ± 250 58%	589 ± 305 52%	10.2 ± 6.0 59%

**Table 2:** Total weight of the study plants with their respective percentage by weight of each part (leaves, stems and roots).

		Total Plant Weight (g)	Plant Part Weight as Percentage of Total (%)		
			Leaves	Stems	Roots
*Miconia* sp.	NZ	10.7 ± 3.6	39.96 ± 0.92	39.7 ± 4.7	20.3 ± 5.6
	PZ	1.96 ± 0.27	60.0 ± 1.3	23.5 ± 1.9	16.5 ± 2.1
*E. polymnioides*	NZ	52 ± 11	17.1 ± 2.3	75.7 ± 1.8	7.23 ± 0.74
	PZ	18.6 ± 2.9	25.5 ± 5.6	56 ± 12	18.4 ± 7.1

**Table 3:** Bioaccumulation and translocation factors for the two study species in a natural zone (NZ) and polluted zone (PZ) subjected to artisanal mining in southern Ecuador.

		Cd		Pb		Zn		Hg	
		NZ	PZ	NZ	PZ	NZ	PZ	NZ	PZ
Bioaccumulation factor (BCF)									
*Miconia* sp.	Leaves	3.47	3.82	0.14	0.23	1.11	0.99	2.05	1.66
	Stems	3.08	2.91	0.12	0.20	0.87	0.78	1.03	0.57
	Roots	3.09	6.50	0.20	0.49	1.48	2.34	1.10	1.05
*E. polymnioides*	Leaves	2.88	6.19	0.25	0.53	4.48	3.28	0.73	1.19
	Stems	2.85	4.62	0.12	0.38	4.53	2.08	0.17	0.31
	Roots	2.42	5.64	0.55	0.80	1.71	1.52	0.76	1.12
Translocation factor (TF)									
*Miconia* sp.	Leaves/roots	1.14	0.84	0.75	0.65	1.00	0.73	2.53	1.72
	Stems/roots	1.00	0.59	0.65	0.50	0.70	0.44	0.87	0.36
*E. polymnioides*	Leaves/roots	1.27	1.55	0.46	0.53	2.40	2.21	1.04	1.27
	Stems/roots	1.08	1.07	0.23	0.43	2.79	1.45	0.25	0.41

## Data Availability

Not applicable.

## Supplementary Materials

The following supporting information can be downloaded at: https://www.mdpi.com/article/10.3390/plants11091186/s1, Table S1: Estimated marginal mean (EMM, also known as least-squares mean) and 95% confidence interval (CI) of Cd, Pb, Zn and Hg concentrations in two species of woody plants native to the Ecuadorian Amazon (*Miconia* sp. and *Erato polymnioides*) as a function of zone (natural versus polluted) and plant part (leaves, stems, roots). EMMs and CIs of each factor (e.g., zone or plant part) were averaged over the levels of the other factor. Pairwise comparisons using Tukey tests are shown for significant effects of zone and/or plant part based on linear models that were executed separately for each metal and species. Differences between zones or plant parts were considered significant if the 95% CI did not include zero (shown in bold). EMMs for non-significant effects (NS) are not shown.

## References

[1] Mahugija J., Kasenya Z., Kilulya K. (2020). Variations of Concentrations of Lead, Zinc, Iron, Copper and Cadmium in Urine of Primary School Pupils in Relation to Age, Sex and Academic Performance. Tanzan. J. Sci..

[2] Mng’ong’o M., Munishi L., Ndakidemi P., Blake W., Comber S., Hutchinson T. (2021). Toxic metals in East African agro-ecosystems: Key risks for sustainable food production. J. Environ. Manag..

[3] Emmanuel A., Jerry C., Dzigbodi D. (2018). Review of Environmental and Health Impacts of Mining in Ghana. J. Health Pollut..

[4] Obiri S., Yeboah P., Osae S., Adu-Kumi S. (2016). Levels of arsenic, mercury, cadmium, copper, lead, zinc and manganese in serum and whole blood of resident adults from mining and non-mining communities in Ghana. Environ. Sci. Pollut. Res..

[5] Buxton A. (2013). Responding to the Challenge of Artisanal and Small-Scale Mining Sustainable Markets.

[6] Chamba I., Rosado D., Kalinhoff C., Thangaswamy S., Sánchez-Rodríguez A., Gazquez M. (2017). Erato polymnioides—A novel Hg hyperaccumulator plant in ecuadorian rainforest acid soils with potential of microbe-associated phytoremediation. Chemosphere.

[7] Alvarez-Berríos N., Aide T. (2015). Global demand for gold is another threat for tropical forests. Environ. Res. Lett..

[8] Stoffersen B., Appel P., Na-Oy L., Sekamane A., Ruiz I., Kóster-Rasmussen R. (2018). Introduction of Mercury-Free Gold Extraction to Small-Scale Miners in the Cabo Delgado Province in Mozambique. J. Health Pollut..

[9] Yeleliere E., Cobbina S., Duwiejuah A. (2018). Review of Ghana’s water resources: The quality and management with particular focus on freshwater resources. Appl. Water Sci..

[10] López-Blanco C., Collahuazo L., Torres S. (2015). Mercury Pollution in Soils from the Yacuambi River (Ecuadorian Amazon) as a Result of Gold Placer Mining. Bull. Environ. Contam. Toxicol..

[11] Wieczorek J., Baran A., Urbański K., Mazurek R., Klimowicz-Pawlas A. (2018). Assessment of the pollution and ecological risk of lead and cadmium in soils. Environ. Geochem. Health.

[12] Rama K., Nishita O., Sadiqa A., Sonal B. (2021). A review on heavy metal contamination at mining sites and remedial techniques. IOP Conf. Ser. Earth Environ. Sci..

[13] Xin J., Ma S., Li Y., Zhao C., Tian R. (2020). Pontederia cordata, an ornamental aquatic macrophyte with great potential in phytoremediation of heavy-metal-contaminated wetlands. Ecotoxicol. Environ. Saf..

[14] Salas-Moreno M., Marrugo-Negrete J. (2020). Phytoremediation potential of Cd and Pb-contaminated soils by Paspalum fasciculatum Willd. ex Flüggé. Int. J. Phytoremediation.

[15] Gupta R., Mohapatra H. (2003). Microbial biomass: An economical alternative for removal of heavy metals from waste water. Indian J. Exp. Biol..

[16] Sardar U., Bhargavi E., Devi I., Bhunia B., Tiwari O. (2018). Advances in exopolysaccharides based bioremediation of heavy metals in soil and water: A critical review. Carbohydr. Polym..

[17] Ashraf S., Ali Q., Zahir Z., Asghar H. (2019). Phytoremediation: Environmentally sustainable way for reclamation of heavy metal polluted soils. Ecotoxicol. Environ. Saf..

[18] Emenike C., Jayanthi B., Agamuthu P., Fauziah S. (2018). Biotransformation and removal of heavy metals: A review of phytoremediation and microbial remediation assessment on contaminated soil. Environ. Rev..

[19] Li S., Zhao B., Jin M., Hu L., Zhong H., He Z. (2020). A comprehensive survey on the horizontal and vertical distribution of heavy metals and microorganisms in soils of a Pb/Zn smelter. J. Hazard. Mater..

[20] Hasan M., Uddin M., Ara-Sharmeen I., Alharby H., Alzahrani Y., Hakeem K., Zhang L. (2019). Assisting Phytoremediation of Heavy Metals Using Chemical Amendments. Plants.

[21] Yan A., Wang Y., Tan S., Mohd-Yusof M., Ghosh S., Chen Z. (2020). Phytoremediation: A promising approach for revegetation of heavy metal-polluted land. Front. Plant Sci..

[22] Favas P., Pratas J., Varun M., Souza R., Paul M. (2014). Phytoremediation of Soils Contaminated with Metals and Metalloids at Mining Areas: Potential of Native Flora. Environmental Risk Assessment of Soil Contaminatio.

[23] Wei Z., Van L., Peng W., Yang Y., Yang H., Gu H., Lam S., Sonne C. (2021). A review on phytoremediation of contaminants in air, water and soil. J. Hazard. Mater..

[24] Baker A. (1981). Accumulators and excluders-strategies in the response of plants to heavy metals. J. Plant Nutr..

[25] Ali H., Khan E., Sajad M. (2013). Phytoremediation of heavy metals--concepts and applications. Chemosphere.

[26] Kachenga L., Chabwela H., Mwauluka K. (2020). Phytoremediation Potential of Indigenous Plants Growing at Nchanga Mine in Chingola, Zambia. Open J. Ecol..

[27] Nkansah F., Belford E. (2017). Heavy Metals Accumulation by Indigenous Plants Growing in Contaminated Soil in a Gold Mining Area in Ghana. J. Nat. Sci. Res..

[28] Tariq F., Samsuri A., Karam D., Aris A. (2016). Phytoremediation of Gold Mine Tailings Amended with Iron-Coated and Uncoated Rice Husk Ash by Vetiver Grass (*Vetiveria zizanioides* (Linn.) Nash). Appl. Environ. Soil Sci..

[29] Surbhi S. (2018). Difference between Standard Deviation and Standard Error (with Comparison Chart)—Key Differences. https://keydifferences.com/difference-between-standard-deviation-and-standard-error.html.

[30] Norma de Calidad Ambiental del Recurso Suelo y Criterios de Remediación Para Suelos Contaminados. Anexo 2 Libro VI 1. https://www.dspace.espol.edu.ec/bitstream/123456789/6078/39/LIBRO%20VI%20Anexo%202%20Remediacion%20de%20suelos.pdf.

[31] Ramírez M., Ramosa J., Angélica R., Brabob E. (2002). Assessment of Hg-contamination in soils and stream sediments in the mineral district of Nambija, Ecuadorian Amazon (example of an impacted area affected by artisanal gold mining). Appl. Geochem..

[32] Anda-Aguirre A. (1986). Zamora de Quito y el oro de nambija.

[33] Chamba I., Gazquez M.J., Selvaraj T., Calva J., Toledo J.J., Armijos C. (2016). Selection of a suitable plant for phytoremediation in mining artisanal zones. Int. J. Phytoremediation.

[34] Nurcholis M., Yudiantoro D.F., Haryanto D., Mirzam A. (2017). Heavy metals distribution in the Artisanal gold mining area in Wonogiri. Indones. J. Geogr..

[35] Faz A., Zornoza R., Muñoz M., Acosta J. (2013). Metals and metalloids in primary gold mining districts of Western Bolivia: Anthropogenic and natural sources. Environ. Earth Sci..

[36] Hasnaoui S.E., Fahr M., Keller C., Levard C., Angeletti B., Chaurand P., Triqui Z.E.A., Guedira A., Rhazi L., Colin F. (2020). Screening of native plants growing on a Pb/Zn mining area in eastern Morocco: Perspectives for phytoremediation. Plants.

[37] Dan-Badjo T., Ibrahim O., Guéro Y., Morel J., Feidt C., Echevarria G. (2019). Impacts of artisanal gold mining on soil, water and plant contamination by trace elements at Komabangou, Western Niger. J. Geochem. Explor..

[38] Fernández S., Poschenrieder C., Marcenò C., Gallego J., Jiménez-Gámez D., Bueno A., Afif E. (2017). Phytoremediation capability of native plant species living on Pb-Zn and Hg-As mining wastes in the Cantabrian range, north of Spain. J. Geochem. Explor..

[39] Basri U., Sakakibara M., Sera K. (2020). Mercury in soil and forage plants from artisanal and small-scale gold mining in the bombana area, Indonesia. Toxics.

[40] Wu B., Peng H., Sheng M., Luo H., Wang X., Zhang R., Xu F., Xu H. (2021). Evaluation of phytoremediation potential of native dominant plants and spatial distribution of heavy metals in abandoned mining area in Southwest China. Ecotoxicol. Environ. Saf..

[41] Gabrielli G., Rodella A., Aparecida C., Coscione A. (2010). Vegetable species for phytoextraction of boron, copper, lead, manganese and zinc from contaminated soil. Sci. Agric..

[42] Liu Z., Chen B., Wang L., Urbanovich O., Nagorskaya L., Li X., Tang L. (2020). A review on phytoremediation of mercury contaminated soils. J. Hazard. Mater..

[43] Susarla S., Medina V.F., McCutcheon S.C. (2002). Phytoremediation: An ecological solution to organic chemical contamination. Ecol. Eng..

[44] Kobina A., Marschner B., Antoniadis V., Stemn E., Shaheen S., Rinklebe J. (2021). Human health risk via soil ingestion of potentially toxic elements and remediation potential of native plants near an abandoned mine spoil in Ghana. Sci. Total Environ..

[45] Mariwy A., Manuhutu J.B., Frans D. (2021). Bioaccumulated Mercury by Several Types of Plants in Ex-Traditional Gold Processing Area, Gogorea Village, Buru Island. Indones. J. Chem. Res..

[46] Xun Y., Feng L., Li Y., Dong H. (2017). Mercury accumulation plant Cyrtomium macrophyllum and its potential for phytoremediation of mercury polluted sites. Chemosphere.

[47] Antonkiewicz J., Kowalewska A., Mikołajczak S., Kołodziej B., Bryk M., Spychaj-Fabisiak E., Babula J. (2022). Phytoextraction of heavy metals after application of bottom ash and municipal sewage sludge considering the risk of environmental pollution. J. Environ. Manag..

[48] Nikolić M., Stevović S. (2015). Family Asteraceae as a sustainable planning tool in phytoremediation and its relevance in urban areas. Urban For. Urban Green..

[49] Sacher W. (2011). Revisión Crítica Parcial del Estudio de Impacto Ambiental para la Fase de Beneficio del Proyecto Minero de Cobre Mirador de la Empresa Ecuacorriente.

[50] Raj D., Chowdhury A., Maiti S. (2017). Ecological risk assessment of mercury and other heavy metals in soils of coal mining area: A case study from the eastern part of a Jharia coal field, India. Hum. Ecol. Risk Assess..

[51] Santoro A., Held A., Linsinger T., Perez A., Ricci M. (2017). Comparison of total and aqua regia extractability of heavy metals in sewage sludge: The case study of a certified reference material. Trends Anal. Chem..

[52] Ashraf S., Tapia W., Villamarín-Ortiz A. (2020). Verificación del método analítico de espectroscopía de absorción atómica con horno de grafito para la cuantificación de cadmio en almendra de cacao (Theobroma cacao). La Granja.

[53] Welna M., Pohl P. (2017). Potential of the hydride generation technique coupled to inductively coupled plasma optical emission spectrometry for non-chromatographic As speciation. J. Anal. At. Spectrom..

[54] Usman K., Al-Ghouti M., Abu-Dieyeh M. (2019). The assessment of cadmium, chromium, copper, and nickel tolerance and bioaccumulation by shrub plant Tetraena qataranse. Sci. Rep..

[55] Sulaiman A.F.R., Hamzah H.A. (2018). Heavy metals accumulation in suburban roadside plants of a tropical area (Jengka, Malaysia). Ecol. Processes.

[56] Shi X., Zhang X., Chen G., Chen Y., Wang L., Shan X.Q. (2011). Seedling growth and metal accumulation of selected woody species in copper and lead/zinc mine tailings. J. Environ. Sci..

[57] Zuur A., Leno E., Walker N., Saveliev A., Smith G. (2009). Mixed Effects Models and Extensions in Ecology with R.

[58] Pinheiro J., Bates D., R Core Team (2022). nlme: Linear and Nonlinear Mixed Effects Models. R Package Version 3.1-157. https://CRAN.R-project.org/package=nlme.

[59] Lenth R. (2022). Emmeans: Estimated Marginal Means, Aka Least-Squares Means. R Package Version 1.7.2. https://CRAN.R-project.org/package=emmeans.

[60] R Core Team R: A Language and Environment for Statistical Computing. https://www.r-project.org/.

