# Supplementary material for: Native Hyperaccumulator Plants with Differential Phytoremediation Potential in an Artisanal Gold Mine of the Ecuadorian Amazon
Source: Plants (Basel). 2022 Apr 28;11(9):1186. doi: 10.3390/plants11091186 (PMC9099852; doi:10.3390/plants11091186)
Supplement: Supplementary file 1 [file plants-11-01186-s001.zip › plants-1627959-supplementary.pdf]

**Supplementary Table S1.** Estimated marginal mean (EMM, also known as least-squares mean) and 95% confidence interval (CI) of Cd, Pb, Zn and Hg concentrations in two species of woody plants native to the Ecuadorian Amazon (*Miconia* sp. and *Erato polymnioides*) as a function of zone (natural versus polluted) and plant part (leaves, stems, roots). EMMs and CIs of each factor (e.g., zone or plant part) were averaged over the levels of the other factor. Pairwise comparisons using Tukey tests are shown for significant effects of zone and/or plant part based on linear models that were executed separately for each metal and species. Differences between zones or plant parts were considered significant if the 95% CI did not include zero (shown in bold). EMMs for non-significant effects (NS) are not shown.

| **Metal** | **Species** | **Trans-formation** | **EMM (± 95% CI)** | | **Pairwise difference (± 95% CI)** | **EMM (± 95% CI)** | | | **Pairwise difference (± 95% CI)** | | |
|---|---|---|---|---|---|---|---|---|---|---|---|
| | | | **Natural** | **Polluted** | **Natural-Polluted** | **Leaves** | **Stems** | **Roots** | **Leaves-Stems** | **Leaves-Roots** | **Stems-Roots** |
| **Cd** | *Miconia* | natural log | 1.07 (0.213, 1.92) | 2.59 (1.76, 3.43) | **-1.53 (-2.63, -0.422)** | NS | NS | NS | NS | NS | NS |
| | *Erato* | none | 1.92 (1.47, 2.36) | 20.57 (8.17, 33.0) | **-18.7 (-31.1, -6.25)** | NS | NS | NS | NS | NS | NS |
| **Pb** | *Miconia* | natural log | 4.16 (3.9, 4.43) | 4.67 (4.24, 5.10) | **-0.507 (-0.978, -0.036)** | NS | NS | NS | NS | NS | NS |
| | *Erato* | none | NS | NS | NS | 84.7 (56.4, 113) | 51 (15.8, 86.2) | 153 (69.5, 236) | 33.7 (-30.0, 97.4) | -68.3 (-177, 40.3) | -102 (-208, 4.29) |
| **Zn** | *Miconia* | none | 70.5 (39.6, 101) | 335.3 (183, 487) | **-265 (-418, -111)** | 210 (129, 292) | 174 (89.6, 259) | 224 (142, 306) | 36.1 (-59.3, 132) | -13.6 (-99.6, 72.4) | -49.7 (-145, 45.7) |
| | *Erato* | natural log | NS | NS | NS | 6.38 (5.94, 6.82) | 6.51 (6.09, 6.93) | 5.60 (5.18, 6.01) | -0.128 (-0.824, 0.567) | **0.784 (0.089, 1.48)** | **0.912 (0.23, 1.60)** |
| **Hg** | *Miconia* | none | 0.762 (0.050, 1.47) | 3.17 (1.25, 5.09) | **-2.41 (-4.38, -0.434)** | 2.53 (1.31, 3.75) | 1.24 (-0.092, 2.57) | 2.13 (0.913, 3.35) | 1.29 (-0.747, 3.32) | 0.393 (-1.47, 2.25) | -0.896 (-2.93, 1.14) |
| | *Erato* | natural log | -0.689 (-0.974, -0.403) | 1.04 (0.541, 1.53) | **-1.73 (-2.26, -1.19)** | 0.605 (0.174, 1.04) | -0.784 (-1.19, -0.377) | 0.702 (0.294, 1.11) | **1.39 (0.681, 2.10)** | -0.097 (-0.805, 0.611) | **-1.49 (-2.18, -0.796)** |
